# Radical Prostatectomy versus External Beam Radiotherapy for cT1-4N0M0 Prostate Cancer: Comparison of Patient Outcomes Including Mortality

**DOI:** 10.1371/journal.pone.0141123

**Published:** 2015-10-27

**Authors:** Satoru Taguchi, Hiroshi Fukuhara, Kenshiro Shiraishi, Keiichi Nakagawa, Teppei Morikawa, Shigenori Kakutani, Yuta Takeshima, Hideyo Miyazaki, Tetsuya Fujimura, Tohru Nakagawa, Haruki Kume, Yukio Homma

**Affiliations:** 1 Department of Urology, Graduate School of Medicine, The University of Tokyo, Tokyo, Japan; 2 Department of Radiology, The University of Tokyo Hospital, Tokyo, Japan; 3 Department of Pathology, Graduate School of Medicine, The University of Tokyo, Tokyo, Japan; Taipei Medical University, TAIWAN

## Abstract

**Background:**

Although radical prostatectomy (RP) and external beam radiotherapy (EBRT) have been considered as comparable treatments for localized prostate cancer (PC), it is controversial which treatment is better. The present study aimed to compare outcomes, including mortality, of RP and EBRT for localized PC.

**Methods:**

We retrospectively analyzed 891 patients with cT1-4N0M0 PC who underwent either RP (*n* = 569) or EBRT (*n* = 322) with curative intent at our single institution between 2005 and 2012. Of the EBRT patients, 302 (93.8%) underwent intensity-modulated radiotherapy. Primary endpoints were overall survival (OS) and cancer-specific survival (CSS). Related to these, other-cause mortality (OCM) was also calculated. Biochemical recurrence-free survival was assessed as a secondary endpoint. Cox proportional hazards model was used for multivariate analysis.

**Results:**

Median follow-up durations were 53 and 45 months, and median ages were 66 and 70 years (*P* <0.0001), in the RP and EBRT groups, respectively. As a whole, significantly better prognoses of the RP group than the EBRT group were observed for both OS and CSS, although OCM was significantly higher in the EBRT group. There was no death from PC in men with low and intermediate D’Amico risks, except one with intermediate-risk in the EBRT group. In high-risk patients, significantly more patients died from PC in the EBRT group than the RP group. Multivariate analysis demonstrated the RP group to be an independent prognostic factor for better CSS. On the other hand, the EBRT group had a significantly longer biochemical recurrence-free survival than the RP group.

**Conclusions:**

Mortality outcomes of both RP and EBRT were generally favorable in low and intermediate risk patients. Improvement of CSS in high risk patients was seen in patients receiving RP over those receiving EBRT.

## Introduction

In 2012, more than 1,112,000 patients worldwide were estimated to be diagnosed with prostate cancer (PC), resulting in more than 307,000 deaths [1]. In Japan, PC is the fourth most commonly diagnosed cancer in men, with an estimated incidence of 51,534 cases (11.8% among 437, 787 cancer patients of all primary sites) in 2008 [2], and that of approximately 9,800 deaths annually when M/I (the number of mortality /number of incidence) of PC is 0.19 [2].

Radical prostatectomy (RP) and external beam radiotherapy (EBRT) are considered as comparable treatments for localized PC [3–11]; however no final consensus exists as to which treatment is better. Furthermore, techniques for both modalities have been evolving rapidly. Laparoscopic and robotic surgery for RP are widely used, with improved outcomes and fewer complications; whereas intensity-modulated radiotherapy (IMRT) for EBRT, which enables increased prostate radiation dose and reduced toxicity in surrounding tissues, has been superseding the conventional method, three-dimensional conformal radiotherapy (3D-CRT) [7]. Therefore, treatment optimization for localized PC should be updated accordingly.

Several studies have compared biochemical recurrence rate of RP and EBRT, most of which have demonstrated their comparability [3–5]. However, we are aware of only two studies that have compared mortality of RP and EBRT, both of which have shown no significant difference [7,8]. Although some epidemiological studies demonstrated lower mortality for RP than EBRT, they analyzed data between the 1990s and the early 2000s [9–11]. More recently, Sooriakumaran et al. conducted large observational study with long follow-up using Swedish population-based dataset between 1996 and 2010, which also suggested that surgery might lead to better survival than radiotherapy [12]. In this context, the present retrospective study aimed to compare outcomes, including mortality, of RP and EBRT for localized PC, based on recent data of consecutive patients treated at our facility.

## Materials and Methods

### Patients

**Fig 1:**
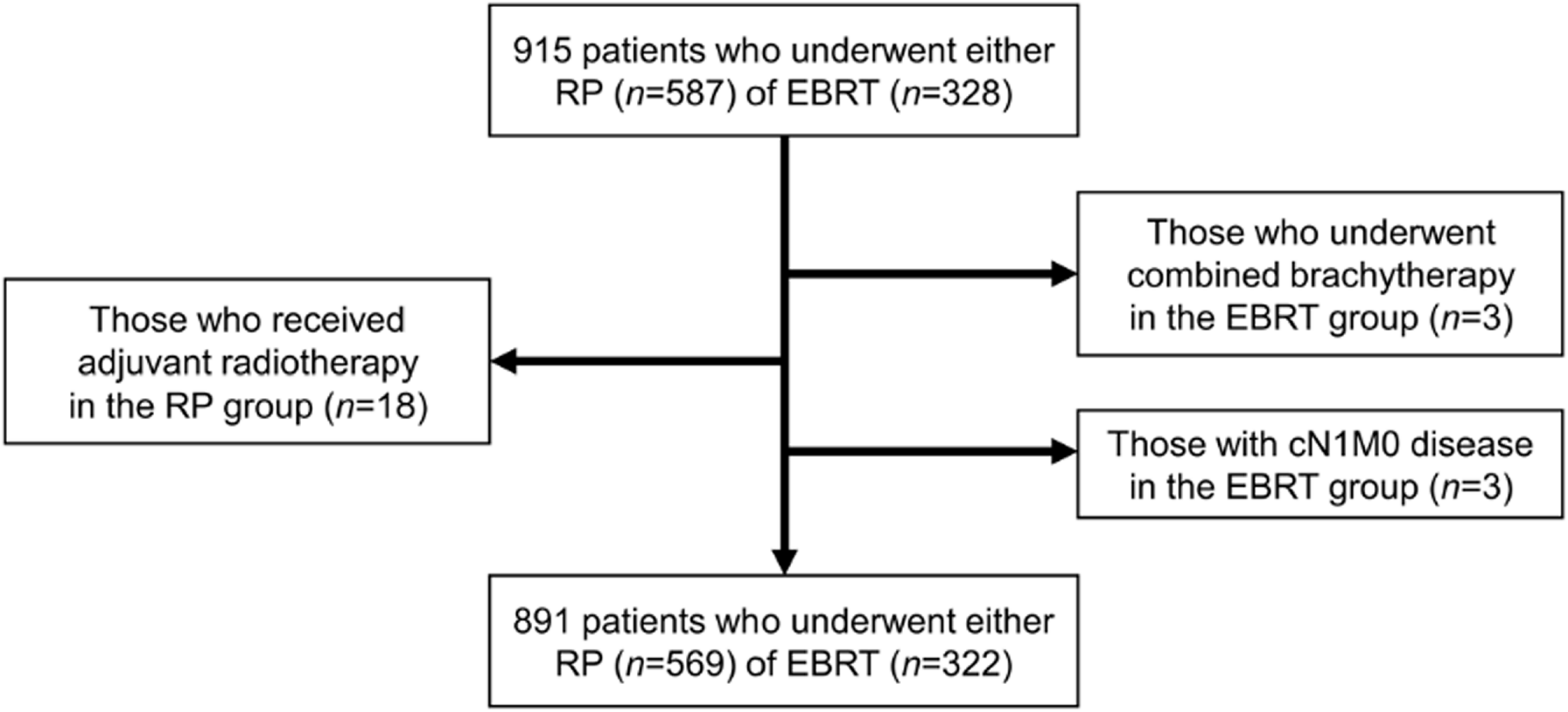
Flow chart representing the study selection process.

We reviewed 915 patients with PC who underwent either RP (*n* = 587) or EBRT (*n* = 328) with curative intent at our single institution between 2005 and 2012. This retrospective study was approved by the institutional review board and was conducted in accordance with the Declaration of Helsinki. We excluded 18 patients who received adjuvant radiotherapy following surgery from the RP group; and three who underwent combination with brachytherapy and three with cN1M0 disease from the EBRT group. Eventually, we retrospectively analyzed 891 patients with cT1-4N0M0 PC who underwent either RP (*n* = 569) or EBRT (*n* = 322) (Fig 1). Patients were stratified according to D’Amico’s risk classification [3]. Charlson comorbidity index (CCI) was assessed for the EBRT group [13], but no information was available for the RP group.

### Treatments

Treatment selection of RP or IMRT was decided by the patient with the referring physician, following an explanation of therapeutic alternatives. Poor surgery candidates and those who preferred radiotherapy to surgery were referred to EBRT. The most common surgical procedure was open RP with bilateral obturator lymph node dissection, which was done on 491 (86.3%) patients. Laparoscopic RP and robot-assisted laparoscopic RP were performed on 36 (6.3%) and 42 (7.4%) patients, respectively.

Most EBRT patients (302 of 322, 93.8%) underwent IMRT, and the remaining few (20 of 322, 6.2%) received 3D-CRT. Most patients (308 of 322, 95.7%) received total doses of ≥72Gy with a median dose of 76 Gy. For the IMRT technique, the vast majority of patients (289 of 302, 95.7%) underwent volumetric modulated arc therapy (VMAT) in which an adequately modulated X-ray beam was delivered to target from a rotating radiation source under computerized optimization to avoid critical organs with inverse planning. Two patients were given VMAT delivery of 26 Gy as a boost following pelvic lymph node irradiation of 45 Gy using fixed four-field (box) technique. The remaining 11 patients received fixed five-field technique with rod compensators developed in our radiotherapy unit [14]. For the 3D-CRT technique, nine patients (45%) received two-axis dynamic arc therapy alone, in which concave dose distribution to avoid bladder and rectum was achieved, for a total dose of 72 Gy [15]; 11 patients (55%) underwent box technique for pelvic lymph node irradiation of 45–50.4 Gy with target boost of 14.4–27 Gy by multi-field technique.

### Endpoints and statistical analysis

Primary endpoints were overall survival (OS) and cancer-specific survival (CSS). Related to these, other-cause mortality (OCM) was also calculated, which was equal to the difference between OS and CSS. Biochemical recurrence-free survival (BRFS) was assessed as a secondary endpoint. Follow-up time started from the day of surgery in the RP group or from the start of radiotherapy for the EBRT group. Biochemical recurrence was defined as two consecutive prostate-specific antigen (PSA) levels of ≥0.2 ng/ml in the RP group [16,17] and the nadir of + ≥2 ng/ml in the EBRT group [18]. Differences in clinical variables between groups were tested using the Student’s *t*- and Chi-square tests.

The OS, CSS, and BRFS were analyzed both globally and according to D’Amico risk for each group. The Kaplan–Meier method was used and log-rank test to compare curves. Cox proportional hazard regression model was used for multivariate analysis. Analysis was performed using JMP Pro version 11.0.0 (SAS Institute, Cary, NC, USA). *P* <0.05 was considered significant. Patients were followed up until December 2014.

## Results

### Patient characteristics

The median (interquartile range [IQR]) follow-up durations were 53 (29–76) and 45 (32–59) months in the RP and EBRT groups, respectively. Table 1 summarizes the clinical characteristics of the patients in both groups. The median (IQR) age was 66 years (62–70 years) and 70 years (66–74 years) in the RP and EBRT groups, respectively (*P* < 0.0001). According to the D’Amico classification [3], 27.6, 42.5, and 29.9% of patients in the RP group; and 13.0, 35.4, and 51.6% of patients in the EBRT group were classified as low-, intermediate-, and high-risk, respectively (*P* < 0.0001). Androgen deprivation therapy (ADT) was more common in the EBRT group than in the RP group (69.3% vs. 23.6%, respectively; *P* <0.0001). In the EBRT group, all patients who underwent ADT received neo-adjuvant ADT followed by its continuation if indicated. In the RP group, of 134 cases who underwent ADT, 107 (79.9%), 18 (13.4%) and 9 (6.7%) used adjuvant ADT, neo-adjuvant ADT and both, respectively.

**Table 1:** Baseline patient characteristics of the RP and EBRT groups in all patients (*n* = 891).

Parameter	RP (*n* = 569)	EBRT (*n* = 322)	*P*-value
Age, years, median (IQR)	66 (62–70)	70 (66–74)	<0.0001* ^a^
Initial PSA, ng/ml, median (IQR)	8.3 (5.8–12.0)	9.7 (6.4–16.9)	<0.0001* ^a^
GS at biopsy, no. (%):			<0.0001* ^b^
≤6	244 (42.9)	66 (20.5)	
7	247 (43.4)	152 (47.2)	
≥8	78 (13.7)	104 (32.3)	
cT, no. (%):			<0.0001* ^b^
1	353 (62.0)	142 (44.1)	
2	168 (29.5)	117 (36.3)	
3	43 (7.6)	60 (18.6)	
4	1 (0.2)	3 (0.9)	
Unknown	4 (0.7)	0 (0)	
Clinical risk group, no. (%):			<0.0001* ^b^
Low	157 (27.6)	42 (13.0)	
Intermediate	242 (42.5)	114 (35.4)	
High	170 (29.9)	166 (51.6)	
ADT, no. (%):			<0.0001* ^b^
Yes	134 (23.6)	223 (69.3)	
No	435 (76.5)	99 (30.7)	
Follow-up duration, months (IQR)	53 (29–76)	45 (32–59)	0.0014* ^a^

RP, radical prostatectomy; EBRT, external beam radiotherapy; IQR, interquartile range; PSA, prostate-specific antigen; GS, Gleason score; ADT, androgen deprivation therapy.

*Statistically significant

^a^Student’s *t*-test

^b^Chi-square test.

Surgical patients were followed with PSA; if biochemical failure was diagnosed, salvage radiotherapy or salvage ADT was given at an appropriate time. Among 569 patients in the RP group, 98 (17.2%) had biochemical failure, of whom 24, 60, and 7 received salvage radiotherapy, salvage ADT, and both, respectively. On the other hand, 21 of 322 (6.5%) in the EBRT group had biochemical failure and all of them received salvage ADT.

Of the 322 patients who underwent EBRT, 99 (30.7%) presented comorbidities, the most common being diabetes mellitus 2, preceding other malignancies and coronary heart disease. All patients who underwent EBRT had age-adjusted CCI ≥1 with a median score of 4 (IQR: 3–4). Furthermore, at least 34 (10.3%) patients in the EBRT group took anticoagulants or antiplatelet agents. CCI was not calculated for the RP group because the information needed was not available.

### Treatment outcomes

**Fig 2:**
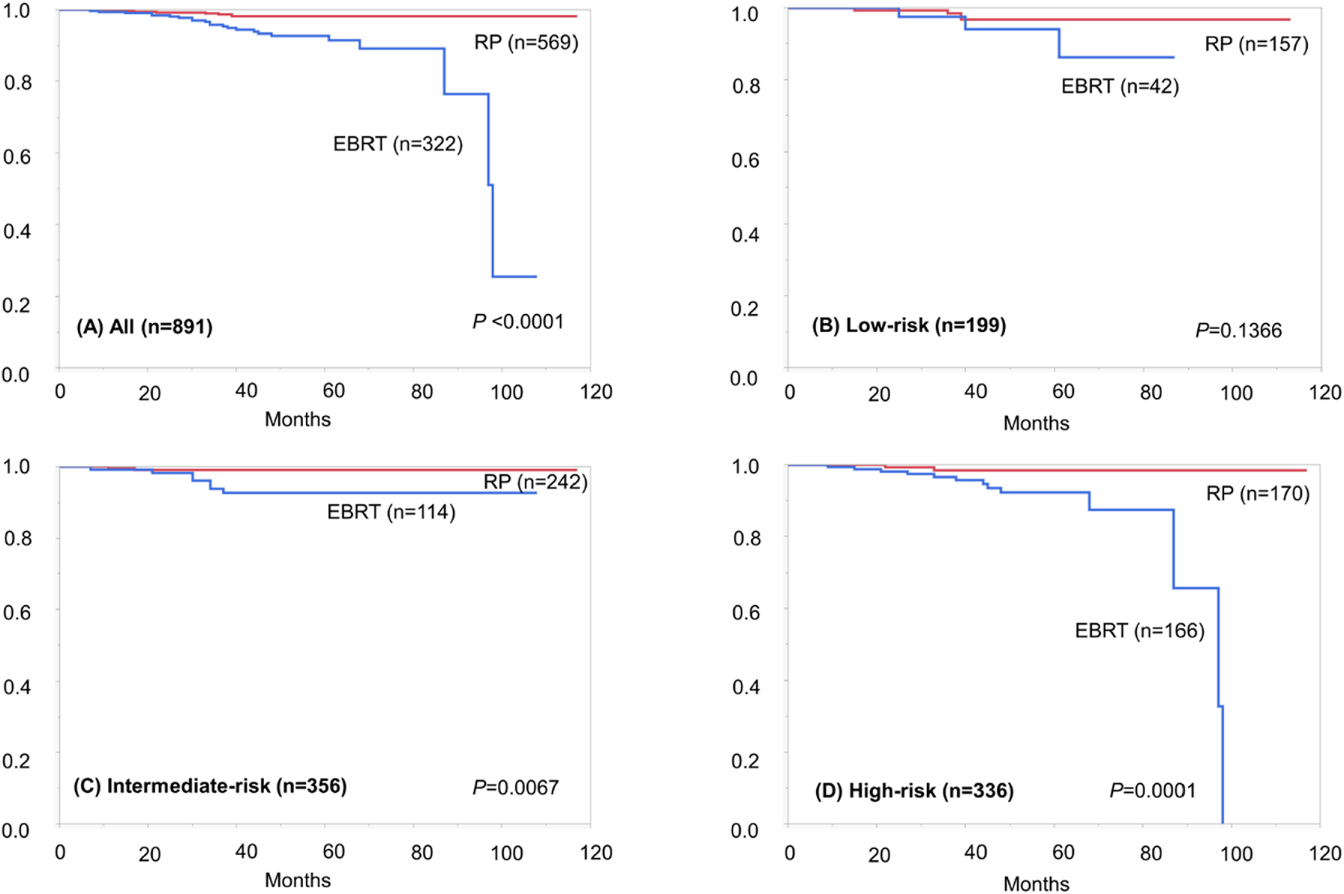
Survival curves depicting overall survival in the radical prostatectomy (RP) and external beam radiotherapy (EBRT) groups, in (A) all (*P* <0.0001), (B) low-risk (*P* = 0.1366), (C) intermediate-risk (*P* = 0.0067), and (D) high-risk patients (*P* = 0.0001), respectively. Each *P*-value indicates the result of log-rank test.

**Fig 3:**
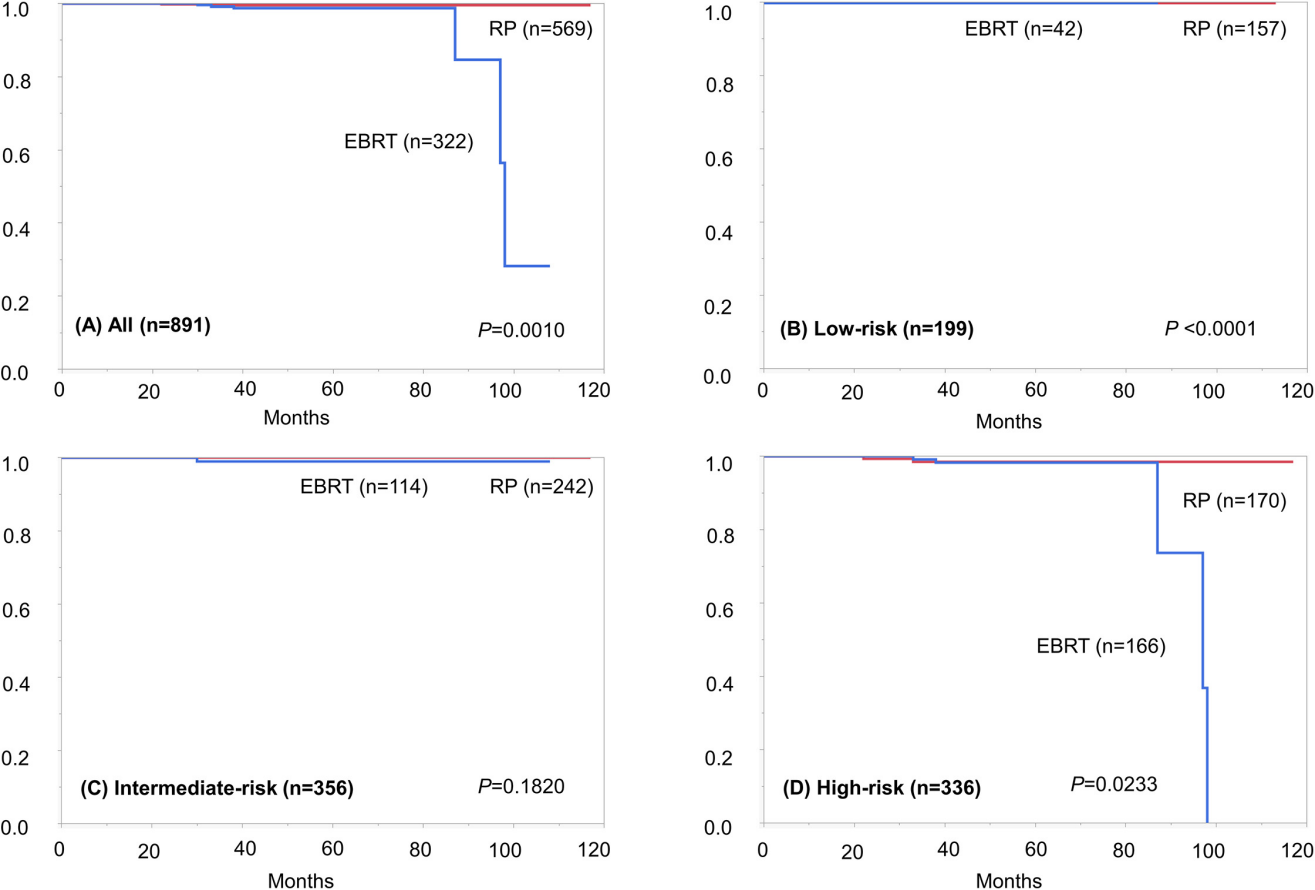
Survival curves depicting cancer-specific survival in the radical prostatectomy (RP) and external beam radiotherapy (EBRT) groups, in (A) all (*P* = 0.0010), (B) low-risk (*P* <0.0001), (C) intermediate-risk (*P* = 0.1820), and (D) high-risk patients (*P* = 0.0233), respectively. Each *P*-value indicates the result of log-rank test.

**Fig 4:**
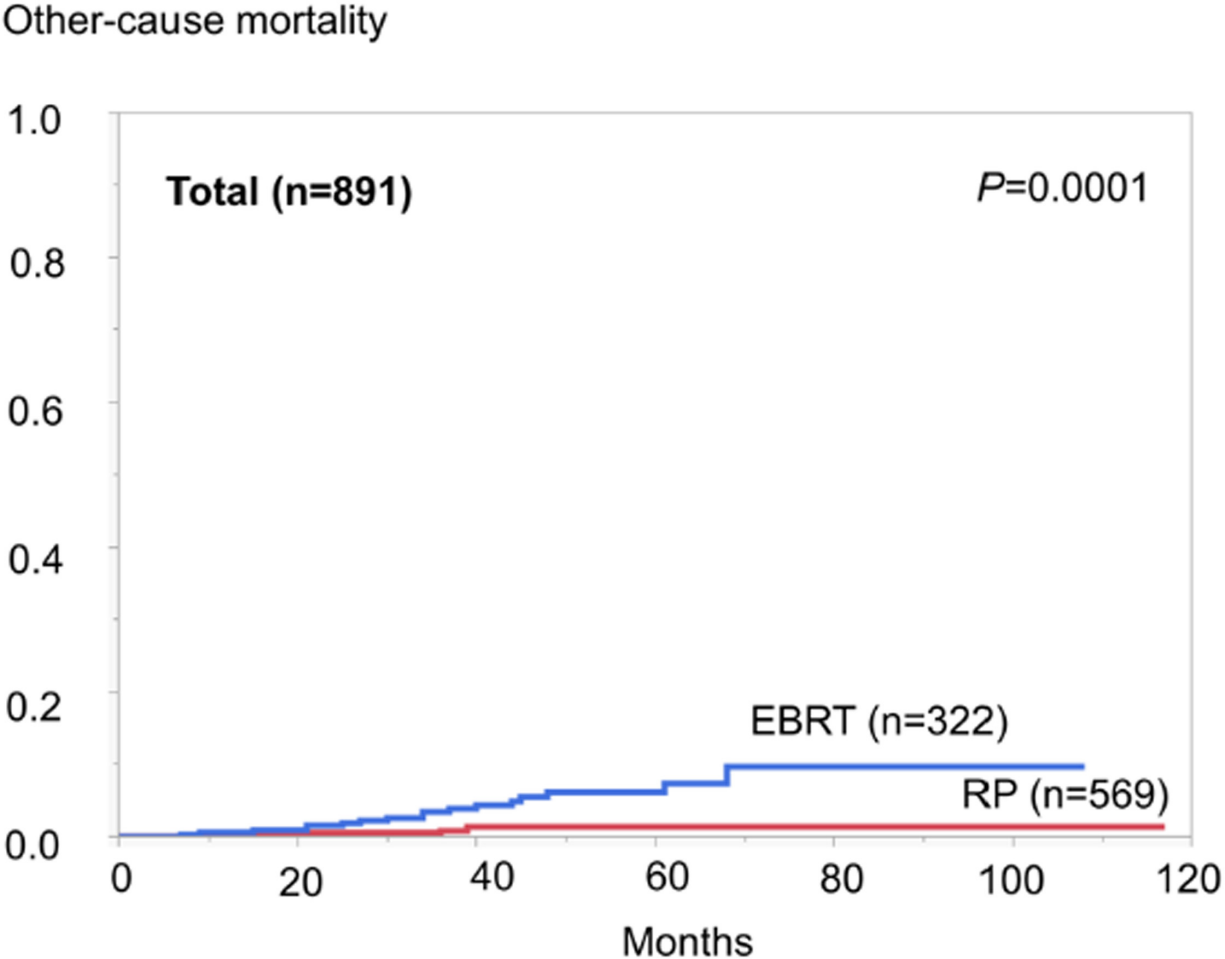
Other-cause mortality in the radical prostatectomy (RP) and external beam radiotherapy (EBRT) groups (log-rank test, *P* = 0.0001).

With regard to the primary endpoints, a total of 31 (3.5%) patients died overall (the RP group: 8; the EBRT group: 23), and 8 (0.9%) died from PC (the RP group: 2; the EBRT group: 6) during the study period. The 5-year OS rates were 98.2 and 92.7%; and 5-year CSS rates were 99.5 and 98.8%, for the RP and EBRT groups, respectively. Survival curves (Figs 2 and 3) indicated a significantly better prognosis of the RP group than the EBRT group, for both OS (log-rank test, *P* <0.0001) and CSS (*P* = 0.0010). Fig 4 shows OCM in the RP group and EBRT group, which shows that the OCM was higher in the EBRT group (*P* = 0.0001). For reference, there were 23 other-cause deaths, and the causes consisted of other types of cancer (*n* = 11: esophageal cancer, *n* = 2; lung cancer, *n* = 2; pancreas cancer, *n* = 2; gastric cancer, *n* = 1; colon cancer, *n* = 1; liver cancer, *n* = 1; bile duct cancer, *n* = 1; and gastrointestinal stromal tumor, n = 1); pneumonia (*n* = 3); cerebral infarction (*n* = 2); aortic dissection (*n* = 1); abdominal aortic aneurysm (*n* = 1); cardiac amyloidosis (*n* = 1); multiple organ failure (*n* = 1); and unknown (*n* = 3).

Concerning CSS in greater detail, there was no death in men with low and intermediate D’Amico risks, except one with intermediate-risk in the EBRT group. In high-risk patients, the superiority of RP over EBRT was demonstrated for both OS (log-rank test, *P* = 0.0001) and CSS (*P* = 0.0233). Related to this, baseline characteristics of the RP and EBRT groups in high-risk patients (*n* = 336) are shown in Table 2. As with the total cohort, there were statistically significant differences of each parameter between the both groups, but lessened differences were observed except age and ADT. Interestingly, of eight patients dying from PC, five died within 38 (range: 22–38) months, while the other three died after 87 (range: 87–98) months. Four in the former group had Gleason score ≥9, while two in the latter had Gleason score 7. Regarding the progression pattern, all patients in the former group initially developed bone metastasis, while each of three in the latter initially developed local recurrence, lung metastasis and pelvic lymph node metastasis, respectively. All three cases in the latter group belonged to the EBRT group.

**Table 2:** Baseline patient characteristics of the RP and EBRT groups in high-risk patients (*n* = 336).

Parameter	RP (*n* = 170)	EBRT (*n* = 166)	*P*-value
Age, years, median (IQR)	66 (62–71)	71 (67–74)	<0.0001* ^a^
Initial PSA, ng/ml, median (IQR)	13.1 (7.3–26.2)	14.8 (7.6–37.8)	0.0188* ^a^
GS at biopsy, no. (%):			0.0005* ^b^
≤6	33 (19.4)	11 (6.6)	
7	59 (34.7)	51 (30.7)	
≥8	78 (45.9)	104 (62.7)	
cT, no. (%):			0.0111* ^b^
1	48 (28.2)	25 (15.1)	
2	76 (44.7)	78 (47.0)	
3	43 (25.3)	60 (36.1)	
4	1 (0.6)	3 (1.8)	
Unknown	2 (1.2)	0 (0)	
ADT, no. (%):			<0.0001* ^b^
Yes	69 (40.6)	153 (92.2)	
No	101 (59.4)	13 (7.8)	
Follow-up duration, months (IQR)	50 (28–71)	44 (31–56)	0.0484* ^a^

RP, radical prostatectomy; EBRT, external beam radiotherapy; IQR, interquartile range; PSA, prostate-specific antigen; GS, Gleason score; ADT, androgen deprivation therapy.

*Statistically significant;

^a^Student’s *t*-test;

^b^Chi-square test

Univariate analysis associated Gleason score at biopsy, clinical T stage, D’Amico’s clinical risk group and treatment modality with CSS. Multivariate analysis of these parameters showed the RP group to be an independent prognostic factor for longer CSS (hazard ratio [HR]: 6.373, *P* = 0.0294) (Table 3). We also analyzed focused on 322 patients in the EBRT group. As with other parameters (age, initial PSA, Gleason score at biopsy, clinical T stage, and D’Amico’s clinical risk group), no significant difference was observed between 3D-CRT and IMRT for both OS and CSS (*P* = 0.5362 for OS and *P* = 0.6330 for CSS). Instead, univariate analysis associated age-adjusted CCI with OS (*P* = 0.0049). In this regard, patients with age-adjusted CCI ≥4 (201 of 322, 62.4%) had significantly higher OCM than those with age-adjusted CCI <4 (121 of 322, 37.6%; *P* = 0.0024).

**Table 3:** Univariate and multivariate analyses of clinicopathological factors for cancer-specific survival.

Parameter	Cutoff	Univariate	Multivariate	
		*P*	HR (95% CI)	*P*
Age	<70 years	0.7208		
	≥70 years			
Initial PSA	≤20 ng/mL	0.1341		
	>20 ng/mL			
GS at biopsy	<8	0.0023*	Reference	0.3878
	≥8		2.037 (0.420 to 14.60)	
cT	<T3	0.0001*	Reference	0.2065
	≥T3		2.899 (0.561 to 21.26)	
Clinical risk group	Low and intermediate	0.0033*	Reference	0.4419
	High		2.877 (0.180 to 73.13)	
Treatment modality	RP	0.0010*	Reference	0.0294*
	EBRT		6.373 (1.195 to 50.25)	

HR, hazard ratio; CI, confidence interval; PSA, prostate-specific antigen; GS, Gleason score; RP, radical prostatectomy; EBRT, external beam radiotherapy.

*Statistically significant.

**Fig 5:**
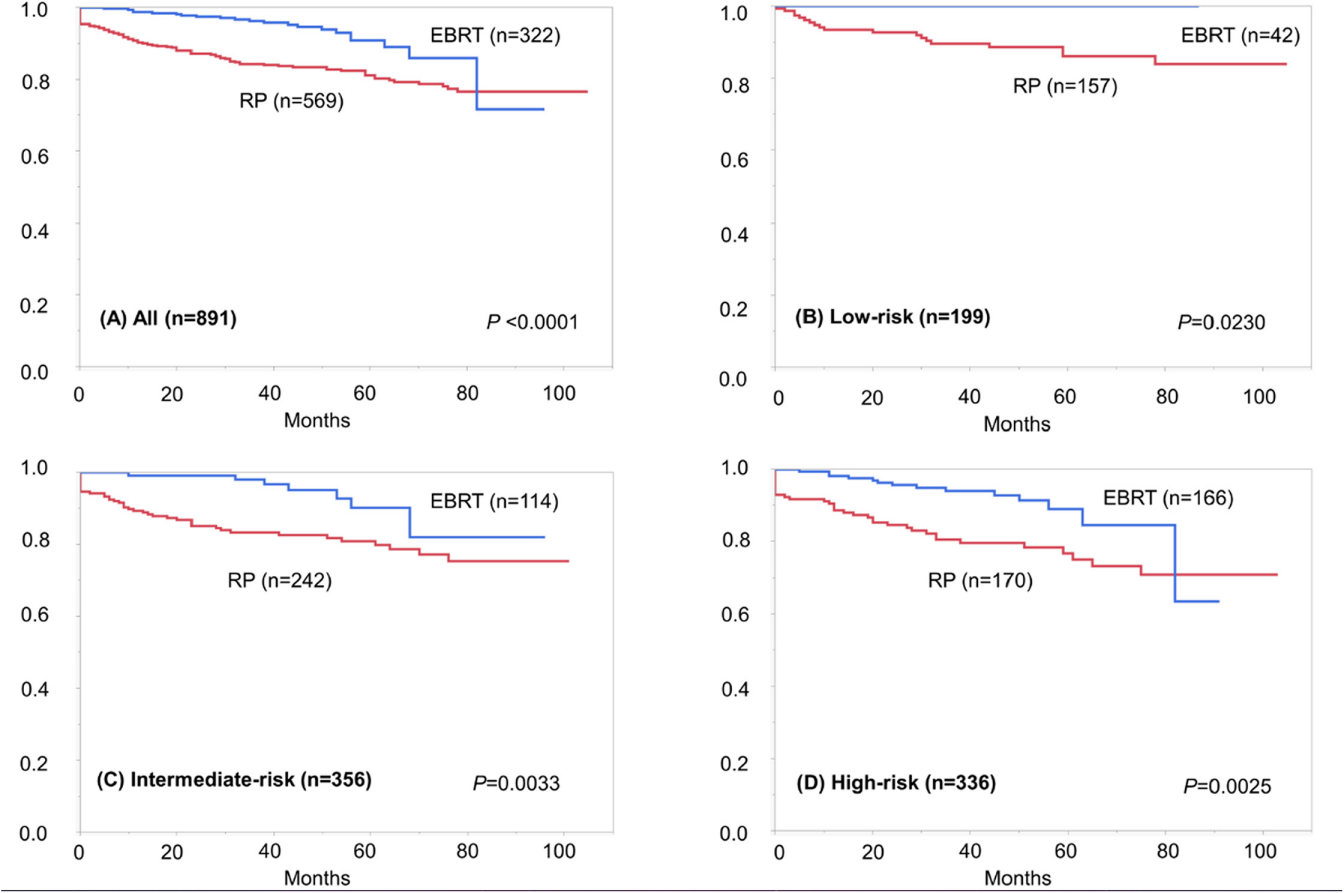
Survival curves depicting biochemical recurrence-free survival in the radical prostatectomy (RP) and external beam radiotherapy (EBRT) groups, in (A) all (*P* <0.0001), (B) low-risk (*P* = 0.0230), (C) intermediate-risk (*P* = 0.0030), and (D) high-risk patients (*P* = 0.0025), respectively. Each *P*-value indicates the result of log-rank test.

On the other hand, the 5-year BRFS rates were 81.1 and 90.9% in the RP and EBRT groups, respectively. The EBRT group had a significantly longer BRFS than the RP group (*P* <0.0001), and the superiority of EBRT over RP was consistent in all three risk groups (Fig 5).

## Discussion

In the present study, we have demonstrated a better survival for RP than EBRT in all the patients and the high-risk patients when analyzed according to D’Amico risk classification. Multivariate analysis confirmed RP as an independent prognostic factor for longer CSS, although the EBRT group was older and had a higher OCM rate. Aside from epidemiologic data analyses, this is the first study to demonstrate the survival advantage of RP over EBRT.

To our knowledge, only two retrospective studies have compared mortality of RP and EBRT [7,8]. Merino et al. reviewed 1,200 patients with localized PC treated with RP (*n* = 993) or IMRT (*n* = 207) in a Chilean population. They reported that treatment modality did not affect the CSS, but that the RP group had longer OS due to greater OCM of the IMRT group [7]. Similarly, Kim et al. reviewed 738 patients comprising RP (*n* = 549) and EBRT (*n* = 189) cases, and reported no inferiority of EBRT to RP despite inclusion of more high-risk patients allocated to EBRT. Notably, however, all patients in the EBRT group of this study underwent 3D-CRT, and patients with cN1 disease were included in the analysis (26 in the RP group and 13 in the EBRT group) [8]. Other than these, some epidemiological studies based on relatively old data demonstrated lower mortality for RP than EBRT, as stated above [9–11]. From the Surveillance, Epidemiology, and End Results (SEER) data, Abdollah et al. identified 68,665 patients with localized PC treated with RP or EBRT between 1992 and 2005, and showed that those treated with RP had a better CSS by propensity score-matched analysis [9]. Two other studies also collected data from the SEER program and drew similar conclusions [10,11]. In addition to these, Sooriakumaran et al. have recently conducted large observational study with long follow-up using Swedish population-based dataset between 1996 and 2010. They evaluated both traditional covariate-adjusted HR and HR after propensity score matching for radiotherapy versus surgery, and have concluded that surgery leads to better survival than radiotherapy [12]. Despite the criticism by radiologists that propensity score matching cannot correct for confounders sufficiently when populations are fundamentally different [19], they have claimed that their data are the best available comparison of efficacy regarding survival between the treatments, until future randomized controlled trials draw a final conclusion [20]. The present retrospective study can be positioned as the additional one which supports the Sooriakumaran’s results in the recent population. Related to this, in our EBRT cohort, 93.8% patients underwent IMRT, and 95.7% received total dose of ≥72Gy with a median dose of 76 Gy, which indicates that our cohort reflects the current clinical situation of EBRT in the era of IMRT.

The reason for longer OS and CSS in the RP group than the EBRT group in this study can be explained as follows. Firstly, EBRT group patients were significantly older, and had a median age-adjusted CCI of as high as 4 (IQR: 3–4), resulting in a higher OCM than the RP group ones. This is attributed to the worse OS of EBRT, and probably to its worse CSS as well, given that patients with comorbidities are considered to be vulnerable to PC itself. For reference, many studies associate pretreatment sarcopenia (muscle loss) with worse outcomes in various malignancies, which indicates that patients’ conditions can affect their ability to deal with stress or disease [21]. Secondly, different salvage treatments might affect the survival outcomes of RP and EBRT. Of 98 RP group patients who developed biochemical failure, 91 (92.9%) received either salvage radiotherapy or salvage ADT at an appropriate time, whereas EBRT patients had fewer options, consisting mainly of alternative ADT and cytotoxic chemotherapy. This might be partially attributed to the worse outcome of EBRT. Indeed, three patients who died from PC late (after 87 months) all belonged to the EBRT group, which might suggest that EBRT patients were likely to fail in salvage treatments. Meanwhile, patients who died early (within 38 months) were presumed to have highly aggressive cancer from the beginning: all except one had Gleason score ≥9.

On the other hand, the BRFS superiority of EBRT to RP depends on the difference of definitions of biochemical recurrence in each group. The PSA nadir of + ≥2 ng/ml in the EBRT group [18] is higher than the two consecutive PSA levels of ≥0.2 ng/ml in the RP group [16,17], although 69.3% of EBRT patients used concomitant ADT. Outcomes of BRFS thus might not reflect those of mortalities, as reported in other studies [7,8].

Given the mortality difference of RP and EBRT, we expect this study to add more evidence as to treatment optimization for localized PC in the era of IMRT. However, this study had several limitations. Firstly, it was a retrospective analysis of a single institution with not a long follow-up, resulting in a very few events. In such conditions, our traditional multivariate analysis could not have been fully-justified for the purpose of correcting for confounders when populations between the modalities were so different, as stated above [19]. Secondly, as with other similar studies, this study suffered from the selection bias. For example, at least 34 (10.3%) patients in the EBRT group took anticoagulants or antiplatelet agents, for which such patients might be assigned to EBRT rather than surgery. Accumulations of such biases would have made the big difference between the two groups. Since the result of multivariate analysis for CSS could be changed if it was conducted together with CCI, the present result should be cautiously interpreted. Thirdly, our EBRT cohort included a small proportion of 3D-CRT cases. As new technologies such as IMRT, VMAT, and image-guided radiotherapy have improved EBRT outcomes [22], evaluations of EBRT against RP should be updated often. In fact, we have obtained a promising VMAT outcome. In our recent VMAT study, only two of 200 patients died from PC in the median follow-up duration of 61 months [23]. Randomized prospective studies using the latest technologies with a longer follow-up period are needed to establish compelling evidence.

## Conclusions

Patients treated with RP had better prognosis for both OS and CSS than those treated with EBRT. Although the patients in the EBRT group was older and had a higher OCM rate, multivariate analysis associated RP with longer CSS. RP might be the better choice, especially for high-risk patients.
